# Supplementary material for: Perceptions and experiences of women and providers on barriers and facilitators of quality emergency obstetric and newborn care services in public hospitals of West Shoa Zone, Oromia, Ethiopia: A phenomenological qualitative study
Source: PLoS One. 2026 Jun 3;21(6):e0350555. doi: 10.1371/journal.pone.0350555 (PMC13232834; doi:10.1371/journal.pone.0350555)
Supplement: S1 File — (PDF) [file pone.0350555.s001.pdf]

Annex: A guiding questionnaires for focused group discussions, in-depth interviews and key informant interview on barriers and facilitators of quality EmONC services in public hospitals of west shoa zone.

Information sheet and Consent form

Dear Participant, good morning/afternoon! My name is ____________ and I am a member of research team at institute of health sciences, Wallega University. We are conducting a research project on barriers and facilitators of quality EmONC services in public hospitals of west shoa zone to improve the quality of emergency obstetric care services. You are identified as one who is going discuss with me on the issue and if you are willing to attend I would like to talk with you about your opinion on the barriers and facilitators of quality emergency obstetric care services among women admitted with obstetric emergencies in public hospitals of west shoa zone for 30-60 minutes.

This interview will be recorded for research accuracy and transcription. Your participation in this research is voluntary. You may withdraw your consent and stop participation at any time without penalty. Your idea during this interview will be kept confidential in final reports. Only the researchers will have access to data tagged with organizational sources, which will be stored on a secured server. While there are no direct benefits to you, we hope to gain more knowledge on how to improve quality of health care services; especially emergency obstetric care services. There are no risks associated with this research. Are there any questions about what I have just explained?

Are you willing to participate in this interview? Yes ☐ No ☐

Name of health facility: _____________________ Respondent Code: _____________________

Interviewer name __________________________signature _____________________________

Supervisors name __________________________signature_____________________________

Time started _________________time end _______________Date of interview ____________

I thank you in advance for your willingness to help with this research!

Type I: A guiding questionnaires for focused group discussion with obstetric care providers on barriers and facilitators of quality emergency obstetric care services in public hospitals of west shoa zone.

1. **Socio-demographic characteristics of the study participants involved in FGDs.**

No	Age of respondent	Sex of respondent	Marital status of respondent	Educational status of respondent	Profession of respondent	Experiences of respondents

2. How you look the emergency obstetric and new born care service provisions in your health facilities?
3. How you see the availability and implementation of all signal functions of emergency obstetric and new born care services in your health facilities?

Probe: Provision of all signal functions of emergency obstetric and new born care services provision in your health facilities.

- Do assisted vaginal delivery (by forceps or ventouse) in your facility in the last three months? If No, what are the reasons?
- Do provision of parenteral anticonvulsants for pre-eclampsia /eclampsia in your facility in the last three months? If yes, which medication is available in your facility? If No, what are the reasons?

- Do provision of parenteral antibiotics for pregnancy-related infections in your facility in the last three months? If yes, which medication is available in your facility? If No, what are the reasons?
- Do provision of parenteral uterotonic/oxytocic drugs in your facility in the last three months? If yes, which medication is available in your facility? If No, what are the reasons?
- Do manual removal of placenta for EmONC patients in the last three months? If No, what are the reasons?
- Do removal of retained products of conception in last 3 months? If No, what are the reasons?
- Do Newborn resuscitations service in the last three months? If No, what are the reasons?
- Do cesarean section provision in your facility in the last three months? If No, what are the reasons?
- Do provision of blood transfusions in the last three months? If No, what are the reasons?

4. According to your opinion, what are the major barriers for providing quality emergency obstetric and new born care services for women admitted with obstetric emergencies?

Probes: all barriers for providing quality emergency obstetric and new born care services from:

- o Client related factors?
- o Providers related factors?
- o Health facility-related factors?
- o Community-related factors?

Probes*:* About staffs (skill, behavior, commitment), logistics (any stoke out): equipment, emergency drugs, supplies, infrastructure (electricity, water…), technical assistance, supervision, transport (ambulance), Service fee, user unable to afford, attendant at community.

5. According to your opinion, what are the facilitators for providing the quality EmONC among women admitted with obstetric emergencies?

Probes: all facilitators to provide quality emergency obstetric and new born care services from:

- o Client related factors?
- o Providers related factors?
- o Health facility-related factors?
- o Community-related factors?

6. How do you see the issues of resources (availability of needed health care providers, availability of equipment's and other resources) at health facility to maintain the quality of emergency obstetric and new born care services?
7. In your opinion, what could be done to improve the quality of emergency obstetric and new born care services among women admitted with obstetric emergencies?
8. Do you have any other comments that you would like to add to improve the quality emergency obstetric care services which haven't been addressed?

Thank you very much for your time and the interview!\

Type II: An interview guide questionnaires for in-depth interview on barriers and facilitators of quality emergency obstetric care services among women admitted with selected obstetric emergencies in public hospitals of west shoa zone.

1. Socio-demographic characteristics of the study participants
   - Name of health facility: ______________
   - Respondent Code: _______________
   - Age of respondent: ______________
   - Sex of respondent: _______________
   - Marital status _________________
   - Educational status of respondent:______________
   - Occupational status of respondent: __________________

2. How do you look the quality of emergency obstetric and new born care services? Specifically, the quality of care provided to you?

3. According to your opinion, what are the major barriers for quality emergency obstetric and new born care service for women admitted with obstetric emergencies?

Probes:

- o Client related factors?
- o Providers related factors
- o Health facility-related factors?
- o Community-related factors?
- o Issues related to access and availability of services?

Probes: staffing (skill, behavior, commitment), logistics (any stoke out): equipment, emergency drugs, supplies, infrastructure (electricity, water…), technical assistance, supervision, transport (ambulance), Service fee, user unable to afford, attendant at community.

4. According to your opinion, what are the facilitators for quality emergency obstetric care among women admitted with obstetric emergencies?

Probes:

- Client related factors?
- Providers related factors
- Health facility-related factors?
- Community-related factors?
- Issues related to access and availability of services

5. How do you look the issues of resources (availability of needed health care providers, availability of equipment's and other resources) at health facility to maintain the quality of emergency obstetric and new born care services?

6. In your opinion, what could be done to improve quality of emergency obstetric and new born care among women admitted with obstetric emergencies?

7. Do you have any other comments that you would like to make with regards to contributory factors in providing quality emergency obstetric and new born care which hasn't been addressed?

Thank you very much for your time and the interview!

Type III: Interview guide questionnaires for key informant interview on barriers and facilitators of quality emergency obstetric care services in public hospital of west shoa zone.

1. Socio-demographic characteristics of the study participants
   - Age of respondent: ________________________
   - Sex of respondent:: ________________________
   - Educational status of respondent:______________________
   - Marital status of respondent:______________________
   - Profession of respondent: ___________________________
   - Experiences of respondents:_________________________
   - Position/role in the facility/sector: ___________________________
2. How you look the status of emergency obstetric and new born care service provision in your health facilities?
3. How you see the availability and implementation of all signal functions of emergency obstetric and new born care services in your health facilities?
4. **Probe:** Provision of all signal functions of emergency obstetric and new born care services in your health facilities?
   - Do assisted vaginal delivery (by forceps or ventouse) in your facility in the last three months? If No, what are the reasons?
   - Do provision of parenteral anticonvulsants for pre-eclampsia /eclampsia in your facility in the last three months? If yes, which medication is available in your facility? If No, what are the reasons?
   - Do provision of parenteral antibiotics for pregnancy-related infections in your facility in the last three months? If yes, which medication is available in your facility? If No, what are the reasons?
   - Do provision of parenteral uterotonic/oxytocic drugs in your facility in the last three months? If yes, which medication is available in your facility? If No, what are the reasons?
   - Do manual removal of placenta for EmONC patients in the last three months? If No, what are the reasons?
   - Do removal of retained products of conception in last 3 months? If No, what are the reasons?
   - Do Newborn resuscitations service in the last three months? If No, what are the reasons?

- Do cesarean section provision in your facility in the last three months? If No, what are the reasons?
- Do provision of blood transfusions in the last three months? If No, what are the reasons?

5. How do you look the issues of resources (Availability of inputs, availability of equipment's, availability of trained human resources) at your public health facilities for providing quality of emergency obstetric and new born care services?
6. According to your opinion, what are the major barriers for providing quality emergency obstetric and new born care services for women admitted with obstetric emergencies?

Probes: all barriers related to:

- o Client related factors?
- o Providers related factors
- o Health facility-related factors?
- o Community-related factors?

***Probes**:* staffing (skill, behavior, commitment), logistics (any stoke out): equipment, emergency drugs, supplies, infrastructure (electricity, water…), technical assistance, supervision, transport (ambulance), Service fee, user unable to afford, attendant at community.

7. According to your opinion, what are the facilitators for quality EmONC among women admitted with obstetric emergencies?

Probes: all facilitators related to:

- o Client related factors?
- o Providers related factors
- o Health facility-related factors?
- o Community-related factors?

8. In your opinion, what could be done to improve the quality of emergency obstetric and new born care services among women admitted with obstetric emergencies?

9. Do you have any other comments that you would like to improve the provisions of quality emergency obstetric care which hasn't been addressed?

Thank you very much for your time and the interview!
