# Supplementary material for: Perceptions and experiences of women and providers on barriers and facilitators of quality emergency obstetric and newborn care services in public hospitals of West Shoa Zone, Oromia, Ethiopia: A phenomenological qualitative study
Source: PLoS One. 2026 Jun 3;21(6):e0350555. doi: 10.1371/journal.pone.0350555 (PMC13232834; doi:10.1371/journal.pone.0350555)
Supplement: S2 File — (PDF) [file pone.0350555.s002.pdf]

The summary of data analysis done on barriers and facilitators of quality EmONC services in public hospitals of West Shoa Zone, Central Ethiopia.

S.No	Major themes	Sub-themes	Identified barriers/facilitators
1	Barriers of quality EmONC services	Health facility related barriers	Shortage of medications, equipment and medical supplies
			Shortage of laboratory services in the facility
			Problem of facility infrastructures (rooms, beds, operating tables water supplies and latrine facilities)
			High client case loads
			Non-communication among liaison office during client referral
			Absence of feedback among liaison office on client referral
			Lack of updated training on EmONC services
			Shortage of human power as standards
			Lack of mentoring and supervision activities
			Shortage of oxygen supply and blood in the facility
			Shortage of ambulance in the facility and at community level
			Less commitment of health facility managements
		Health care providers related barriers	Knowledge and skill gaps among HCPs
			Lack of compassionate & respectful maternity care
			Inadequate counseling provision to obstetric clients
			Delays to providing EmONC services
		Client and community related barriers	Gap of community awareness on obstetric emergency cases
			Delays to getting the EmONC services

2	Facilitators of quality EmONC services	Health facility related facilitators	Availability of free maternity services
			Availability of mixed health care providers in the facility (Gynecologists, IESOs, midwives, General practitioners, Nurse)
			Availability of obstetric service every 24hrs
			Establishment of NICU service in the facility
			Establishment of blood bank in the nearby area
			Availability of liaison office for client referral linkages
			Availability of community pharmacy at health facility
		Health care providers related facilitators	Providers commitment and teamwork for maternity care
			Availability of ambulances in the facility
		Client and community related facilitators	Client and community willingness for getting services
			Community support of mothers transportation during referral
