# Supplementary material for: Perceptions and experiences of women and providers on barriers and facilitators of quality emergency obstetric and newborn care services in public hospitals of West Shoa Zone, Oromia, Ethiopia: A phenomenological qualitative study
Source: PLoS One. 2026 Jun 3;21(6):e0350555. doi: 10.1371/journal.pone.0350555 (PMC13232834; doi:10.1371/journal.pone.0350555)
Supplement: Attachment — Submitted filename: Response to Reviewers.docx [file pone.0350555.s004.docx]

**A rebuttal letter for reviewers Questions**

1. **Is the manuscript technically sound, and do the data support the conclusions?**

- Yes, the introduction was trying to identify the gap of the study as supported by reviewers and the study was conducted with a carefully designed research method using a phenomenological qualitative study design among women experienced obstetric emergencies, their obstetric care providers and facility managers to explore the barriers and facilitators of quality emergency obstetric care services. The study also included adequate sample sizes incorporating IDIs, FGDs and KIIs. To ensure the trustworthiness of data, careful sampling and participant selection were performed to provide sufficient information about the research and to assure the transferability of the findings. Finally, an inductive thematic analysis was applied to analysis the data and findings were presented with texts, tables and graphs. Therefore, this scientific research was technically sound and the conclusions was drawn based on the study findings to provide an insight for policy makers to protect maternal morbidity and mortality.

2. **Has the statistical analysis been performed appropriately and rigorously?**

- N/A. Since the study was conducted through a qualitative study design, applying statistical analysis for the study was not applicable. But, we were used the MAXQDA version 24.4.1 software to code and facilitate the data analysis, and an inductive thematic analysis was applied to analyze the data in rigorously manners.

3. **Have the authors made all data underlying the findings in their manuscript fully available?**

- Yes, as both reviewers of this manuscript were supported during their reviewing feedback of this manuscript, all the data of this finding was available and we will also provide all additional needed data when requested.

4. **Is the manuscript presented in an intelligible fashion and written in Standard English?**

- Yes, we tried to write the manuscript in clear, logical and grammatically correct sentences before submitted to the journal. An English expert reviewing was also involved in checking and editing of grammatically errors. Furthermore, we also made some editing in this revision of manuscript based on comments given.

5. **For Reviewers comment**

- Reviewer #1 comments: As the reviewer commented, we look and accommodate all the comments given very critically to make this research work a more impactful article. Therefore, we have gone through all comments and incorporated in the revised manuscript like the issues of strengthening the study gaps including references, sampling method of health facilities to be clear and number of total study participants included and making the graphs included in the manuscript to be more readable and visibly.
- For Reviewer #2 comments: We have gone through all comments given and incorporated in the revised manuscript like the issues of results mentioned under the barriers to provision of EMNOC services and their facilitators, and the need to provide some solutions to the perceived barriers in order to improve the quality of EMNOC provision.

6. Do you want your identity to be public for this peer review?

- NO, because, I need the confidentiality of my research data and other works.
