# Supplementary material for: Perceptions and experiences of women and providers on barriers and facilitators of quality emergency obstetric and newborn care services in public hospitals of West Shoa Zone, Oromia, Ethiopia: A phenomenological qualitative study
Source: PLoS One. 2026 Jun 3;21(6):e0350555. doi: 10.1371/journal.pone.0350555 (PMC13232834; doi:10.1371/journal.pone.0350555)
Supplement: Attachment — Submitted filename: Response_to_Reviewers_auresp_2.docx [file pone.0350555.s005.docx]

**A rebuttal letter responding to each points raised by academic editor and reviewer(s)**

**For Reviewer #1 comments:**

**Answer**

- As indicated in the review comments, the first reviewer has no further comments for the authors and mentioned as all his/her comments are fully addressed by authors.

**For Reviewer #3 comments:**

Q1. The reviewer comments the manuscript title is long and considers shortening of the title for a better impact?

**Answers**

- The author took the comments and made minimizing (shortening) the manuscript title to bring a better impactful research work.

Q2. As indicated in the review comments to the Author, there were grammatical errors and problem of sentences construction throughout the manuscript that compromise the clarity and the manuscript need an improvement and professional language editing before submission.

**Answers**

- Based on the reviewer comments, we made great effort to make the correction of spelling, grammar correction and amendments for all the given comments by reviewer.
- We also involved professional language editor for the correction of grammatical errors based on reviewer recommendations.

Q3. In the method section, the reviewer asked the Author:

- Why were these hospitals chosen? What criteria were used for saturation? How data quality and validity were ensured beyond just "verbatim transcription."

**Answers**

- The included hospitals were chosen purposively based on the obstetric caseloads and the research objectives considering all types of hospitals (primary, general and referral hospitals) found in the study area.
- The specific criteria used for data saturation in this study were checking for saturation (i.e. no new themes emerging during the data collection time and when all conceptual information is explored and completed). It is also ensured when new data consistently repeats the previous information and codes. Furthermore, it is also ensured through including all potential categories of study populations (emergency obstetric mothers, providers, and facility managers) and using multiple data collection methods were applied to secure data saturation.

- In addition to the verbatim transcription, data quality and validity of this study were ensured through audit of all written field notes, recording respondents’ expressions and other data documents. Furthermore, the systematic application including data **credibility, transferability, dependability and conformability were applied effectively to ensure the** data quality and validity**.**

Q4. The reviewer asked the author to write MVA abbreviation fully as first mentioned in the manuscript

**Answers**

- As the reviewer commented, the Author corrected as manual vacuum aspiration in the manuscript as commented.

Q5. The reviewer asked the Author as most references are not up to date (within the last 5 years) and it need to update some of the references

**Answers**

- We have taken this comment as positive feedback for the improvement of manuscript; we tried our best by improving some of the paragraph in the introductory part with their references to increase the impactfulness of the research work as good evidence.

**For Reviewer #4 comments:**

Q1. In the abstract part the reviewer commented about spelling grammatical errors, study design and study aim need to be corrected in the manuscript. The reviewer also commented the Abbreviations should be written in fully first.

**Answers**

- We took this comment as great feedback for the improving this manuscript and the author made significant improvements on the grammatical error correction and all other comments given that needed amendments in the manuscript.
- We also written the abbreviations first in fully at all place and used as commented latter.

Q2. In the introduction part the reviewer commented as there are spelling errors, incorrect tenses and grammatical errors that need amendments

**Answers**

- As commented by reviewer, the author made significant improvements on the spelling, tense and grammatical error correction to make an impactful manuscript.

Q3. In the introduction part the reviewer commented to explain how fetal distress is a cause of neonatal death

**Answer**

- For neonates, the primary causes of death are often related to intra-partum events and maternal conditions, specifically birth asphyxia (related to obstructed/prolonged labor), infections (sepsis), and prematurity.

Q4. In the introduction part the reviewer commented to recasting some sentences, some statements need to be referenced, need to update some references to the more recent ones and explaining the context of some statements and other comments.

**Answers**

- As commented by reviewer, the author made significant improvements on the correction of all comments given to make an impactful research manuscript.

Q5. The reviewer commented to the authors to explain how we arrived the conclusion in line 70 **Answers**

- As commented by reviewer, we made some modification on the sentence that indicates a conclusion of things from the literature finding.

Q5. In the method part the reviewer commented as there are spelling errors, incorrect tenses and grammatical errors that need amendments

**Answers**

- As commented by reviewer, the author made significant improvements on the spelling, tense and grammatical error correction to make an impactful manuscript.

Q6. The reviewers asked how services were/are provided by integrated emergency obstetric surgery or how integrated emergency obstetric surgery was an inclusion criterion

**Answer**

- The word “Integrated emergency obstetric surgery” is indicating those health care providers doing obstetric managements including performing caesarian section in obstetrics and other activities of obstetric management. These Integrated emergency obstetric surgery providers are a registered professional working in obstetric department by federal ministry of health in order to fill the gaps of gynecologist in the country.

Q7. The reviewer commented about why verbal consents were obtained from the participants and not written informed consents which is conventional.

**Answer**

- As reviewer commented both verbal and written informed consents were obtained from the participants. Therefore, we corrected in the manuscript now.

Q8. The authors should explain in detail how sample size was determined, and how they selected their participants and interviewed them.

**Answer**

- As mentioned for the Reviewer #3 above, the sample size was determined using the saturation of data through checking for data saturation (i.e. no new themes emerging during data collection and when all conceptual information is explored and completed) through including all potential study populations and using multiple data collection methods

Q9. In the result part, the reviewer commented the spelling of “gynecology” should be correct

**Answer**

- As commented by reviewer to correct the word, the word gynecology was edited correctly in the manuscript as commented.
