# Supplementary material for: Perceptions and experiences of women and providers on barriers and facilitators of quality emergency obstetric and newborn care services in public hospitals of West Shoa Zone, Oromia, Ethiopia: A phenomenological qualitative study
Source: PLoS One. 2026 Jun 3;21(6):e0350555. doi: 10.1371/journal.pone.0350555 (PMC13232834; doi:10.1371/journal.pone.0350555)
Supplement: Attachment — Submitted filename: Response to Reviewers and Editor letter.docx [file pone.0350555.s006.docx]

**Point-by-point response for comments of Academic editor and reviewers on the manuscript submitted**

**MANUSCRIPT ID: PONE-D-25-03007R2**

#### MANUSCRIPT TITLE: PERCEPTIONS OF WOMEN AND PROVIDERS ON BARRIERS AND FACILITATORS OF QUALITY EMERGENCY OBSTETRIC CARE IN OROMIA, ETHIOPIA

1. **Issues raised by Editors (Editors comments)**

| EDITOR COMMENT | AUTHOR RESPONSE | PAGE NUMBER |
| --- | --- | --- |
| Introduction part |  |  |
| The editor asked the author in introduction part of line 67-72: In the first paragraph, it would be important to incorporate the most recent evidence from the 2023 WHO report. Additionally, please ensure that the maternal mortality ratio for Ethiopia is reported using the latest available data from the recent report to improve the accuracy and relevance of the background information? | The author has taken the given comments of updating to the recent data and it was incorporated in the introductory part of the manuscript. | Page 3 |
| The editor asked the author in introduction part of line 96-104: It is unclear why the discussion relies on the 2016 Ethiopian Demographic and Health Survey (EDHS) report despite the availability of a more recent report. Referring to the latest data would provide more up-to-date evidence and strengthen the relevance of the discussion. | The author has taken the given comments and incorporated in the introductory part of the manuscript with the available findings of a more recent report. | Page 4 |
| The editor asked the author in introduction part of line 105: you mentioned that “The recent evidence shows that half of all maternal and one million newborn deaths can be prevented by providing high-quality care before, during and after childbirth. However, in low-income countries, obstetric mothers receive less than half of the recommended practices in atypical maternity care visit (14)”…how the study published in 2018 become recent? | The author has modifying some terms used in this paragraph and corrected the comments in the edited manuscript. | Page 4 |
| The editor asked the author to clearly justify why a phenomenological approach was chosen to address the research questions. | The author response for why a phenomenological approach chosen to address the research questions is that it is an appropriate design and well suited to exploring individuals’ lived experiences and perceptions of a phenomenon. Additionally, since the quality of care is influenced by personal experiences, interactions, and contextual factors within health facilities, a phenomenological approach allows for an in-depth understanding of these experiences and incorporated in **the study design part of a method section** in the edited manuscript. | Page 6 |
| The editor asked the author, it would be helpful to explain why methodological triangulation was not considered. Alternatively, the authors should clarify whether this study is part of a broader or larger research project. | The author response for this question is this manuscript was not considered for methodological triangulation but it is a part of a larger PhD research project incorporating both the quantitative and qualitative part. The quantitative part was addressed by other independent manuscripts. | Page 5 |
| The editor asked the author in introduction part to include the rationale for selecting the West Shoa zone as the study area should be clearly described. Since the study was conducted only in a limited geographic area, it would be more appropriate to specify West Shoa Zone in the title. | The author took the comments and corrected in the end paragraph of the introduction by including the rationale for selecting the West Shoa zone as the study area and on the title of this manuscript by including West Shoa Zone. | Page 5 |
| Method part | | |
| The editor asked the author to use a reporting guideline and ensure to follow the appropriate reporting guidelines when preparing manuscript and submit the completed checklist as supplementary material. (ensure to follow the appropriate SRQR checklist reporting guidelines) | As the editor commented, all the given comments were incorporated as SRQR checklist reporting guidelines and included in the revised manuscript and also submitted the completed checklist as supplementary material. | Page  6-9 |
| The editor asked the author to state in the methods which guidelines were consulted when preparing the manuscript. In this study, you can utilize a SRQR checklist to organize and report your technique section. | As the editor commented, the SRQR checklist was used to organize and report the manuscript work, and mentioned in study design. | Page 6 |
| The editor asked the author to check your study and source population? You mentioned only women? And what about health care workers? | As the editor commented, all the given comments were incorporated in the method section of the edited manuscript. | Page 7 |
| Result part |  |  |
| The editor asked the author in the results part, themes “Barriers related to health care facilities,” “Barriers related to health care providers,” and “Barriers related to clients and the community as a whole.” However, the specific sub-themes under each theme should be clearly and explicitly presented so that readers can easily understand the structure and meaning of the findings. The same approach should also be applied to the facilitators, where the corresponding sub-themes should be clearly outlined under each main theme. | The author took the given comments and corrected in the result part of this manuscript for both barriers and facilitators of quality emergency obstetric and new-born care services. | Page  12-18 |
| The editor asked the author in the results part, the way the discussants’ statements are presented requires substantial improvement. When quoting health workers, please provide complete information in brackets, such as age, professional field, and years of experience. In addition, the language used should clearly reflect that the statements are directly expressed by the discussants, ensuring that the quotations accurately represent the participants’ voices. | The author incorporated all editor comments for manuscript improvements and the manuscript fully edited to in reach the editor concerns or points. | Page  13-18 |
| Discussion part | | |
| The editor commented in the discussion part, the discussion lacks depth, as it only compares the results across studies. When comparing the study findings with reports from the existing literature, it is recommended to comment on the reasons for the observed discrepancy based on the theoretical framework or give a plausible explanation. It is also required to indicate its clinical and theoretical implications on the topic of interest. | The author incorporated all editor comments for the discussion part to improve the manuscript and the manuscript has fully checked and edited to in reach the editor concerns. | Page  18-21 |
| Strengths and Limitations of Study | | |
| The editor commented the author in the Strengths and Limitations of Study, the limitations should address potential biases (e.g., selection or recall bias), constraints in data collection (e.g., self-reporting or incomplete data), and generalizability of the findings to other populations or settings. | The author incorporated all the limitations that the editor commented for the study including the selection or recall bias, constraints in data collection and generalizability of the study findings to other populations or settings. | Page 22 |
| General comments | | |
| The editor general comment for the author was the manuscript needs English language editing, particularly grammar, comma and tense use. | The author incorporated all editor comments to improve the manuscript and the manuscript has fully checked and edited to in reach the editor concerns. | Page  1-27 |
| The editor general comment for the author was based on your study design, stick to Research Reporting Checklist such as SRQR checklist | The author incorporated the editor comments and the manuscript has fully checked with Research Reporting Checklist of SRQR checklist. | Page  1-27 |
| The editor general comment for the author was checking the references writing and ensure as per journal requirements. | The author incorporated all editor comments related to the references and corrected in the current manuscript. | Page  24-27 |

2. **Issues raised by Reviewers**/**Review Comments to the Author**

| REVIEWERS COMMENT | AUTHOR RESPONSE | | PAGE NUMBER |
| --- | --- | --- | --- |
| A general reviewer 4 comments, for the author is there are still several grammatical errors, poorly constructed sentences, incorrect tenses, incorrect use of plural and singular nouns and spelling errors that need to be addressed. | | As the reviewer commented, the author have critically checked, incorporated and made significant improvement to the current edited manuscript for publication. | Page  1-27 |
| Introduction section | | | |
| In introduction, based on the context of the statement, the reference citing lines 58-60 should be a more recent one, Line 55 should better read -- Obstetric emergencies, and Lines 64-68-- Evidence shows (and the statement should be referenced). | | As the reviewer commented, we have incorporated all the given comments in the currently edited manuscript for publication. | Page 3 |
| In introduction, Abbreviations should 1st be written in full, then in bracket eg EMONC, SDG. Lines. | | As the reviewer commented, we have incorporated all the given comments in the currently edited manuscript for publication. | Page  3-5 |
| In introduction, Sentences in line 78-79 should be referenced. | | As the reviewer commented, we have corrected sentences of the paragraph and incorporated the references in the currently edited manuscript for publication. | Page 4 |
| The method section | | | |
| In the methods, the authors in lines 114-115 should explain why the 4 hospitals were selected. | | As the reviewer commented, we have explained and included why the 4 hospitals were selected from the total hospitals. | Page 6 |
| Several sentences should be re-casted and grammar corrected eg, lines 119-120, 123-124, 132-134, 147-148, | | As the reviewer commented, we have corrected the errors occurring throughout the manuscript | Page 6-9 |
| Spelling errors in the inclusion criteria eg preeclapsia/eclapsia which correctly should be preeclampsia/eclampsia and this should be corrected throughout the manuscript. | | As the reviewer commented, we have corrected the errors occurring throughout the manuscript | Page  1-26 |
| In lines 138=139, how emergency obstetric surgery an obstetric care provider? | | It was corrected as “Integrated Emergency Surgical Officers (IESOs)” - in Ethiopia a task-shifting initiative by the Federal Ministry of Health (FMoH) that trains non-physician clinicians—to perform essential, life-saving surgery. This MSc-level, three-year training allows these professionals to handle obstetric emergencies (e.g., C-sections, uterine rupture) and general surgical trauma at primary hospitals. | Page 7 and 11 |
| Abbreviations eg Gyn should 1^st^ be written in full. | | As the reviewer commented, we have fully written and made improvement in the current manuscript. | Page 7 |
| In lines 154-155, the data instrument refereed to should be referenced. | | As the reviewer commented, we have added the some references of the adapted data instrument for this study in the manuscript. | Page 8 |
| The result section | | | |
| The results have grammatical and spelling errors  eg lines 162, 189, 173, 176. | | As the reviewer commented, we have seen all comments and made correction in the manuscript. | Page  10-18 |
| Abbreviations in the tables should be written under them as footnotes. | | As the reviewer commented, the author written all the Abbreviations in the tables in fully form | Page  11-12 |
| Lines 326-329, 364-367 should be re-casted for clarity. | | As the reviewer commented, we have made improvement the current edited manuscript to clarify grammatically correct more. | 17 |
| Correct error in line 335 and should be corrected. | | As the reviewer commented, we made correction and fully written OB-GYN specialists in the manuscript. | 17 |
| The discussion section | |  |  |
| The discussion has several grammatical errors and wrong use of singular and plural nouns. | | As the reviewer commented, we have made significant improvement in the discussion part of the current edited manuscript to clarify the manuscript grammatically corrected. | Page  19-22 |
| The referencing section | |  |  |
| The referencing style should be uniform and in accordance with that of the journal. The following are either not complete, not well written as journal, numbers 16, 22, 26, 27, 28, 30, 31, 33, 38. | | As the reviewer commented, we have made re-organizing the result into subsections including demographics, obstetric/service factors, input, process, output, regression) in the manuscript. | Page  24-27 |

**NB. Page number giving is according to the file in the Revised Manuscript with Track Changes.**
