# Supplementary material for: Perceptions and experiences of women and providers on barriers and facilitators of quality emergency obstetric and newborn care services in public hospitals of West Shoa Zone, Oromia, Ethiopia: A phenomenological qualitative study
Source: PLoS One. 2026 Jun 3;21(6):e0350555. doi: 10.1371/journal.pone.0350555 (PMC13232834; doi:10.1371/journal.pone.0350555)
Supplement: Attachment — Submitted filename: Response_to_Reviewers_and_Editor_letter_auresp_4.docx [file pone.0350555.s007.docx]

**Point-by-point response for comments of Academic editor and reviewers on the manuscript submitted**

**MANUSCRIPT ID: PONE-D-25-03007R2**

#### MANUSCRIPT TITLE: PERCEPTIONS OF WOMEN AND PROVIDERS ON BARRIERS AND FACILITATORS OF QUALITY EMERGENCY OBSTETRIC CARE IN OROMIA, ETHIOPIA

1. **Issues raised by Reviewers**/**Academic editor to the Author**

| REVIEWER 4 COMMENTS | AUTHOR RESPONSE | | PAGE NUMBER |
| --- | --- | --- | --- |
| A general reviewer 4 comment for the author is that the authors have significantly improved the manuscript and it is now acceptable for publication. | | First, we want to thank all reviewers and editors for their professional work to review our manuscript and gave us significant comments to be improved and get the chance of publication. | All pages |
| Another reviewer #4 comment for the authors is there are just few errors that need to be addressed editorially. Line 121- researcher should be researchers, line 310- carcity should be scarcity, line 346 delivere - deliver. | | As the reviewer #4 commented, the authors have critically checked and incorporated all given editorial comments to made significant improvement to correct the editorial problem in the manuscript for getting publication. | Page 5  Page 13  Page 14 |
| Another reviewer #4 comments given for the authors is lines 479-484, 490-494 and 499-501 should be recasted. | | As the reviewer commented, we have made significant improvement in recasting current manuscript to clarify the manuscript grammatically corrected. | Page  19 - 20 |
| EDITOR COMMENTS | | **AUTHOR RESPONSE** | **Page No** |
| The editor commented the authors as “You stated that “The Ethiopian demographic health survey (EDHS) in 2024-2025 also reported that the current Ethiopian maternal mortality rate and neonatal mortality rate were 169 deaths per 100,000 live births and25 deaths per 1000 live births, respectively” Are you sure that Ethiopian MMR is 169 ? | | Thank you for your great input to correct our mistake about using appropriate terms to write the findings on the issues. As you commented the authors, we have modified to **According to preliminary findings from the Ethiopia Demographic and Health Survey (EDHS) 2024–2025 report**. The preliminary findings are different from Ethiopian demographic health survey finding. | Page 3 |
| The editor also commented the authors as kindly check figure again. | | As editor commented, the authors have checked all given comments and incorporated in the manuscript for getting publication. Furthermore, there was no figure in the manuscript and all the tables have been checked critically. | All pages |

**NB. Page number giving is according to the file in the Revised Manuscript with Track Changes.**
