# Supplementary material for: Perceptions and experiences of women and providers on barriers and facilitators of quality emergency obstetric and newborn care services in public hospitals of West Shoa Zone, Oromia, Ethiopia: A phenomenological qualitative study
Source: PLoS One. 2026 Jun 3;21(6):e0350555. doi: 10.1371/journal.pone.0350555 (PMC13232834; doi:10.1371/journal.pone.0350555)
Supplement: Attachment — Submitted filename: Response to Reviewers and Editor.docx [file pone.0350555.s008.docx]

**Point-by-point response for comments of Academic editor and reviewers on the manuscript submitted**

**MANUSCRIPT ID: PONE-D-25-03007R2**

#### MANUSCRIPT TITLE: PERCEPTIONS OF WOMEN AND PROVIDERS ON BARRIERS AND FACILITATORS OF QUALITY EMERGENCY OBSTETRIC CARE IN OROMIA, ETHIOPIA

1. **Issues raised by Academic editor to the Authors**

| EDITOR COMMENTS | AUTHOR RESPONSE | | PAGE NUMBER |
| --- | --- | --- | --- |
| The editor commented the authors as “You stated that “The Ethiopian demographic health survey (EDHS) in 2024-2025 reported that the current Ethiopian maternal mortality rate and neonatal mortality rate were 169 deaths per 100,000 live births and 25 deaths per 1000 live births, respectively” However, the figure of 169 deaths per 100,000 live births refers to the Pregnancy-Related Mortality Ratio (PRMR), not the Maternal Mortality Ratio (MMR). Standard reporting generally uses the Maternal Mortality Ratio; therefore, this figure should be checked and corrected accordingly. | | Thank you very much for your great input to correct our work and to use appropriate terms (MMR) to write the findings on the issues. Therefore, as you commented the authors, we have made modified to:  **According to preliminary findings from the Ethiopia Demographic and Health Survey (EDHS) of 2024–2025, the maternal mortality ratio (MMR) was 141 deaths per 100,000 live births, and the neonatal mortality rate was 25 deaths per 1000 live births, respectively.** | Page 3 |
| The editor also commented the citation format should follow Vancouver or modified Vancouver style. The current citation does not fully comply with this format and should therefore be revised and properly formatted. | | As editor commented, the authors have checked all citation formats and **corrected as Vancouver style in the revised manuscript** as necessary for getting appropriate writing for publication. | Page  22-24 |

**NB. Page number was given according to the file in the Revised Manuscript with Track Changes.**
